# Aortic Root Thrombus in a Left Ventricular Assist Device Patient as a Cause for Intractable Ventricular Tachycardia

**DOI:** 10.1016/j.atssr.2025.01.004

**Published:** 2025-02-01

**Authors:** Krish C. Dewan, Alejandro A. Lobo, Zachary W. Fitch, Alejandro Murillo, Angela Pollak, Alina Nicoara, Violet G. Johnston, Carmelo A. Milano

**Affiliations:** 1 Department of Surgery, Duke University Medical Center, Durham, North Carolina; 2 Department of Anesthesiology, Duke University Medical Center, Durham, North Carolina

## Abstract

Aortic root thrombosis (ART) is an uncommon complication after left ventricular assist device implantation. We describe a unique postoperative presentation of intractable ventricular tachycardia as a result of an aortic root thrombus extending into the left main coronary artery. This case highlights the importance of a high degree of suspicion and anticoagulation for ART in the setting of intractable postoperative ventricular tachycardia and limited opening of the aortic valve. Second, whereas most reported cases of ART have been managed conservatively by optimizing left ventricular assist device speed and anticoagulation, we demonstrate the feasibility of early surgical management.

Aortic root thrombosis (ART) is an uncommon complication after left ventricular assist device (LVAD) implantation. LVAD patients with end-stage heart failure are at risk for development of lethal ventricular arrhythmias from a number of possible mechanisms.^1^ Here we describe the case of a patient with a unique postoperative presentation of intractable ventricular tachycardia as a result of an aortic root thrombus extending into the left main (LM) coronary artery.

The patient is a 50-year-old man with idiopathic dilated cardiomyopathy and a remote history of cardioembolic stroke. His chronic heart failure deteriorated with need for intravenous inotropic support as well as an intra-aortic balloon pump. He was taken to surgery and underwent implantation of a HeartMate 3 LVAD (Abbott Cardiovascular). The patient was initially anticoagulated with heparin and bridged to warfarin with target international normalized ratio (INR) of 2 to 3. On postoperative day 9, intractable ventricular tachycardia developed with hemodynamic deterioration; he was unresponsive to cardioversion and antiarrhythmia medications. Transthoracic echocardiography revealed a mobile thrombus within the aortic root that was confirmed with computed tomography imaging (Figure 1). He was taken back to the operating room. Intraoperative transesophageal echocardiography confirmed a large thrombus in the root with extension into and obstruction of the LM coronary ostia (Figure 2). The sternal incision was reopened, and cardiopulmonary bypass was reestablished with central cannulation. The LVAD was stopped, the ascending aorta cross-clamped, and the heart arrested with cold del Nido cardioplegia solution delivered in a retrograde manner. An aortotomy was performed, and the clot was removed from the aortic root. However, the cardioplegia solution did not back flush out of the LM coronary ostium. Therefore, a Fogarty balloon-tipped catheter, maximum diameter of 5 mm (Edwards Lifesciences), was introduced into the LM and directed into the circumflex branch. The balloon was partially inflated, and the catheter was withdrawn to remove clot (Figure 3). This was repeated until no further clot was retrieved and retrograde cardioplegia solution could be seen emanating from the ostium. The aortotomy was closed; the heart was reperfused, and a paced rhythm was maintained. A central right ventricular assist device was installed, and the patient was supported for 48 hours. The patient gradually recovered after removal of the right ventricular assist device, with no further ventricular arrhythmias. He was anticoagulated with heparin and then warfarin for a higher INR goal of 2.5 to 3.5 and ultimately discharged to a rehabilitation facility. He was discharged home after 2 weeks and is currently free from any recurrent thrombosis, arrhythmias, or LVAD-related complications at 14 months after discharge. He has been maintained on a home regimen of amiodarone, mexiletine, and warfarin with an INR goal of 2 to 3, has completed physiotherapy, and is currently under evaluation for transplant candidacy. The Duke University institutional review board determined that this study is not research and that its approval was not required.

**Figure 1:**
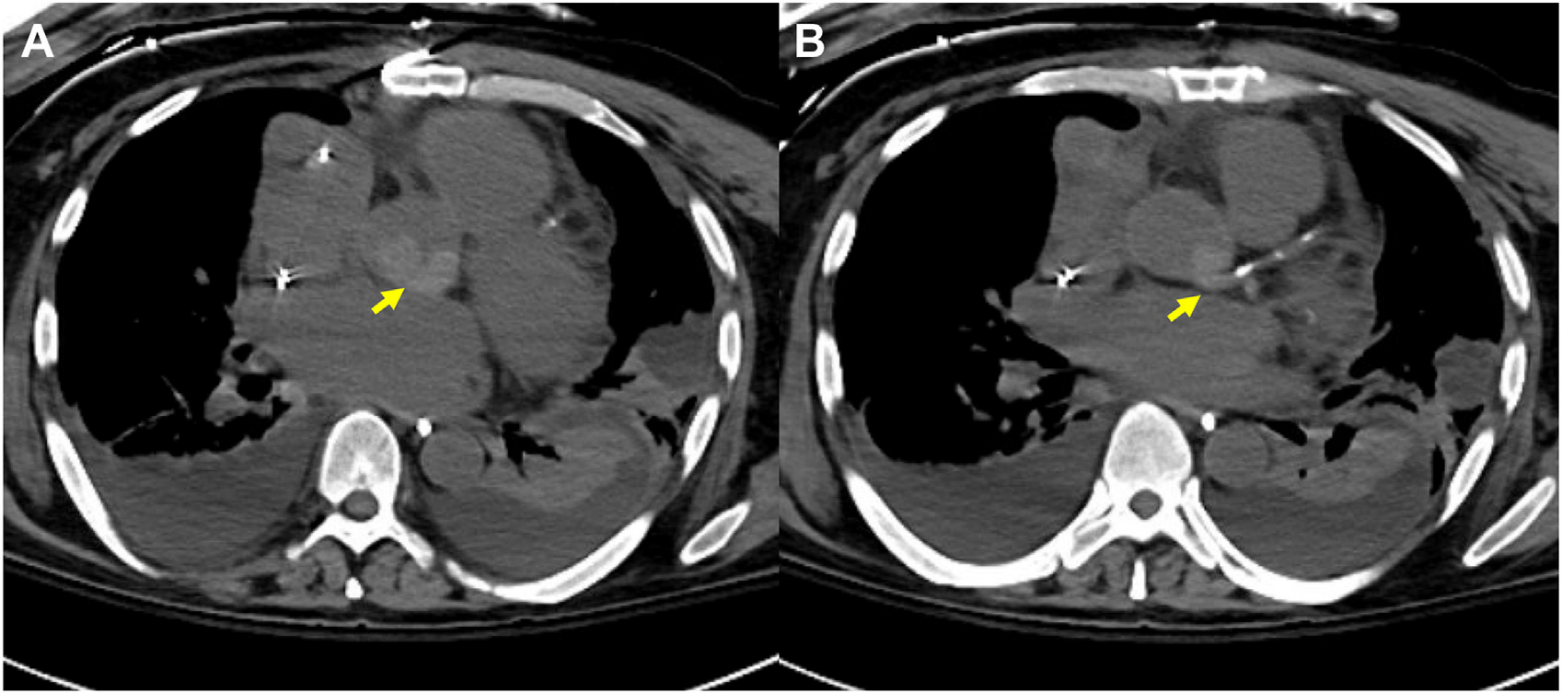
Computed tomography imaging demonstrating (A) a high-density 2.7 × 1.6-cm material within the aortic root (arrow), (B) extending into the proximal left main coronary artery (arrow).

**Figure 2:**
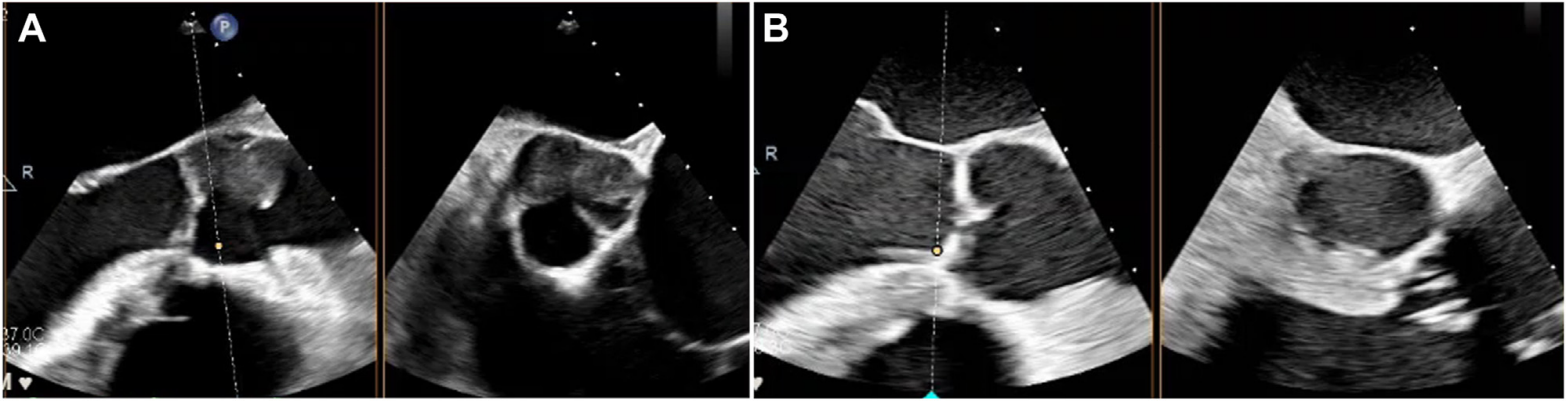
Intraoperative transesophageal echocardiography demonstrating (A) preoperative 2.3 × 2.3-cm thrombus in aortic root along left and noncoronary cusps of the aortic valve with evidence of left coronary artery occlusion and (B) after successful thrombectomy.

**Figure 3:**
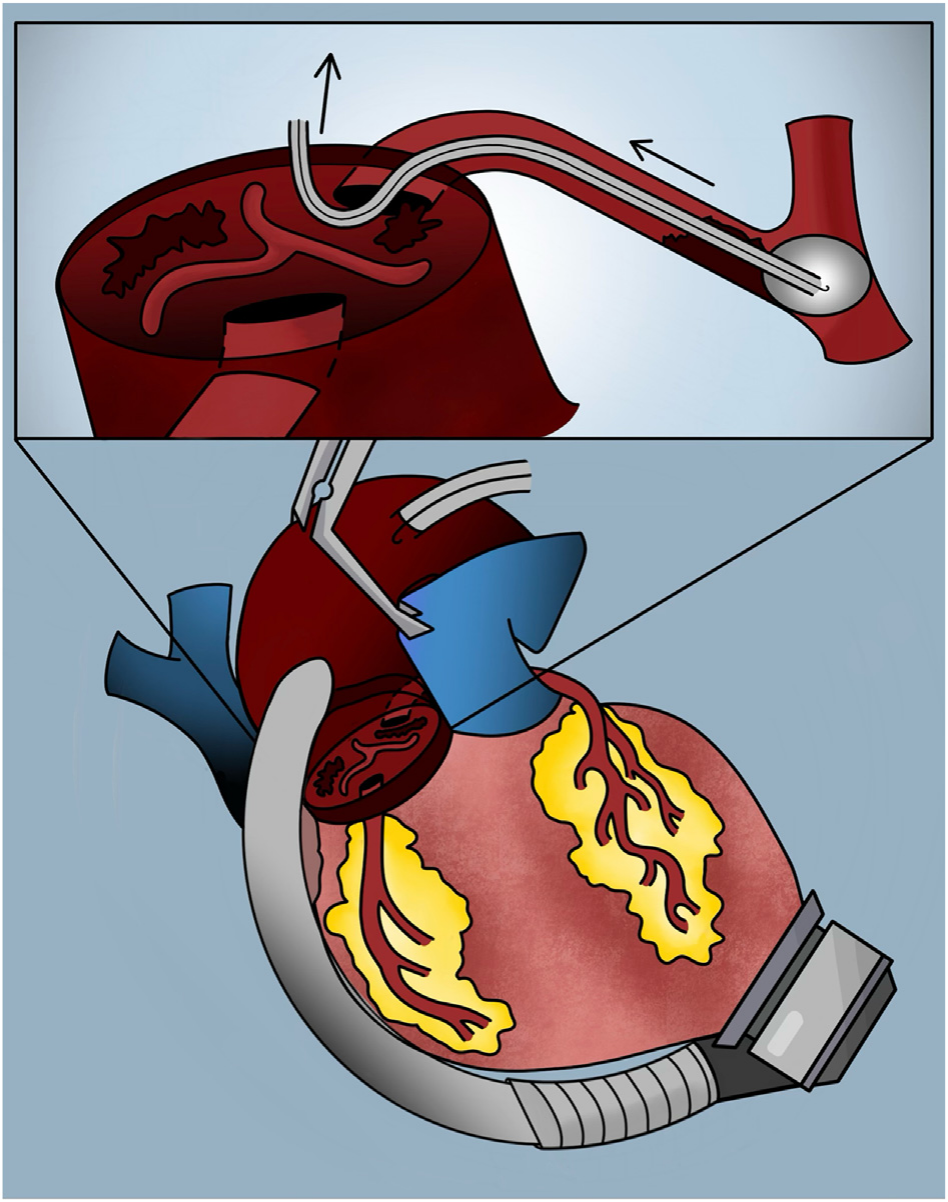
Removal of aortic root and intracoronary thrombus using a Forgarty balloon catheter.

## Comment

Intractable ventricular arrhythmias can occur in LVAD patients with terminal cardiomyopathies. The cause is variable and includes altered ventricular substrate, mechanical irritation from the apical cannula, and electrolyte abnormality. In many cases, the cause is not understood. In this case, a closed aortic valve early after LVAD implantation resulted in reduced flow through the root and thrombus formation. The thrombus occluded the LM ostium, resulting in ischemia manifested as intractable ventricular arrhythmias. Thrombosis of the LM is an unusual presentation, given that thrombosis most often occurs in the noncoronary cusp.^2^ In the absence of other possible factors, ART in the setting of an immobile aortic valve should be considered in the differential for postoperative ventricular tachyarrhythmias.

As in this case, nearly 90% of patients with ART present with an immobile aortic valve.^2^ This case emphasizes the importance of anticoagulation, particularly when there is limited opening of the aortic valve. Early intensification of anticoagulation goals may prevent ART formation and morbidity associated with the sequelae. Whereas most reported cases of ART have been managed conservatively by optimizing LVAD speed and anticoagulation,^2^ this patient required aggressive surgical treatment to remove the thrombus along with Fogarty balloon catheter thrombectomy of the LM coronary to restore the coronary circulation. In previously reported cases of postoperative myocardial infarction secondary to ART treated conservatively, right ventricular failure worsened and was complicated by embolic stroke and death.^3^ Despite these prior reports, we do not advocate for a “surgery-first” approach to managing ART. Rather, when conservative measures fail to address acute decompensation from ART, an expeditious surgical approach to remove the thrombus is feasible and safe, especially in the acute postimplantation period. Given the infrequency of this complication and the surgical approach described, there is no protocolized risk stratification to guide whether surgery or conservative measures are warranted. The choice of surgical vs conservative management is to be judged on a case-by-case basis by the surgeon, given the urgency and clinical context of the presentation. In this case, without intervention, the patient is likely to have died of the resulting refractory arrhythmia and hemodynamic instability. Hence, an expedient surgical approach was warranted despite possible risks of coronary dissection or reperfusion injury.

In conclusion, postoperative ART should be considered a cause for intractable ventricular arrhythmia after LVAD implantation, particularly when the aortic valve is predominantly closed. Early intensification of anticoagulation may be preventive, and early surgical thrombectomy was effective in this case.
